# Multidimensional Response Surface Methodology for the development of a gene editing protocol for p67^phox^-deficient Chronic Granulomatous Disease

**DOI:** 10.1089/hum.2023.114

**Published:** 2024-01-24

**Authors:** Thomas E. Whittaker, Shefta E Moula, Sameer Bahal, Faris Ghalib Bakri, Wail Ahmad Hayajneh, Ammar Khaled Daoud, Asma Naseem, Alessia Cavazza, Adrian J Thrasher, Giorgia Santilli

**Affiliations:** 1 Infection, Immunity and Inflammation Teaching and Research Department, Great Ormond Street Institute of Child Health, University College London, United Kingdom; 2 Division of Infectious Diseases, Department of Medicine, Jordan University Hospital, Amman, Jordan; 3 Infectious Diseases and Vaccine Center, University of Jordan, Amman, Jordan; 4 Division of Infectious Diseases, Department of Pediatrics, Jordan University of Science & Technology, Irbid, Jordan; 5 Division of Immunology, Department of Internal Medicine, Jordan University of Science & Technology, Irbid, Jordan; 6 NIHR Great Ormond Street Hospital Biomedical Research Centre, London, United Kingdom

## Abstract

Replacing a faulty gene with a correct copy has become a viable therapeutic option as a result of recent progress in gene editing protocols. Targeted integration of therapeutic genes in hematopoietic stem cells has been achieved for multiple genes using CRISPR/Cas9 system and Adeno-Associated Virus (AAV) to carry a donor template. Although this is a promising strategy to correct genetic blood disorders, it is associated with toxicity and loss of function in CD34+ hematopoietic stem and progenitor cells, which has hampered clinical application. Balancing the maximum achievable correction against deleterious effects on the cells is critical. However, multiple factors are known to contribute, and the optimisation process is laborious and not always clearly defined.

We have developed a flexible multidimensional Response Surface Methodology approach for optimisation of gene correction. Using this approach, we could rapidly investigate and select editing conditions for CD34+ cells with the best possible balance between correction and cell/CFU loss in a parsimonious one-shot experiment. This method revealed that using relatively low doses of AAV2/6 and CRISPR/Cas9 ribonucleoprotein complex, we can preserve the fitness of CD34+ cells and, at the same time, achieve high levels of targeted gene insertion. We then used these optimised editing conditions for the correction of p67^phox^-deficient chronic granulomatous disease, an autosomal recessive disorder of blood phagocytic cells resulting in severe recurrent bacterial and fungal infections, and achieved rescue of p67^phox^ expression and functional correction of CD34+-derived neutrophils from a CGD patient.

## Introduction

Chronic Granulomatous Disease (CGD) is an inherited primary immunodeficiency in which the NADPH oxidase complex loses some or all function, resulting in the inability of neutrophils and other phagocytic cells to generate sufficient reactive oxygen species (ROS) necessary to kill pathogens after engulfment.^1,2^ As a result, patients experience recurrent and often life-threatening bacterial and fungal infections and suffer from inflammatory complications. The NADPH oxidase comprises five core subunits and one newly discovered chaperone protein (EROS).^3,4^ Mutations in the X-linked gp91^phox^ (encoded by the *CYBB* gene) are the most common aetiology, accounting for around 65% of identified disease. Recessive mutations in the autosomal subunits p22 ^phox^ (*CYBA*), p40 ^phox^ (*NCF4*), p47 ^phox^ (*NCF1*) and p67 ^phox^ (*NCF2*) account for the remaining 35% of disease. Although comparatively rare, autosomal disease is significantly more prevalent in areas of the world in which consanguinity is common, with p67^phox^-deficiency (p67-CGD) being the most prevalent form in Jordan.^5^ X-linked CGD has previously been regarded as the most severe form; however, it is now apparent that both X-linked and autosomal forms can vary in presentation depending on residual NADPH oxidase activity, and so p67^phox^-deficient patients may present with severe disease.^6,7^ Currently, the only curative treatment is allogeneic transplantation of hematopoietic stem/progenitor cells (HSPCs), a procedure that carries significant risks when the patient does not have a good match.^1,7,8^ Genetic correction of X-linked CGD (gp91^phox^) has been intensely investigated with both classical integrating lentiviral treatments^9^ and Clustered Regularly Interspaced Short Palindromic Repeats (CRISPR)/Cas9 gene editing techniques^10,11^, recently leading to successful lentiviral gene therapy clinical trials.^12^ Gene correction has been carried out on some of the autosomal mutations that cause the remaining 35% of disease;^13–15^ however, these variants are comparatively understudied. Biochemical correction of p67-CGD has been previously reported with a retroviral strategy^16^ and by insertion of a *NCF2* minigene into the *AAVS1* locus.^17^ However, retroviral therapies have been found to cause carcinogenic insertional mutagenesis^18^ and insertions outside of native genomic context are not under native regulation. It is important to avoid deregulated expression of any subunit of the NADPH oxidase, as increased ROS production in HSPC could limit their life span and contribute to loss of engraftment.^19,20^ Insertion into the genome of functional p67^phox^ cDNA could be accomplished by non-site-specific integration of a lentiviral construct and promoter, or by site-specific insertion of the cDNA into the native genomic context, allowing control by extant regulatory sequences. Site-specific insertion can be accomplished by making a double-strand breakwith a designer nuclease (Cas9) targeted to a specific location by a single guide RNA and providing a donor template containing cDNA flanked by homology arms *in trans*. The DNA donor template is most typically provided by coinfection with recombinant adeno-associated virus (AAV). Homology-directed repair (HDR) processes repair the breakage using the template to synthesise replacement DNA, inserting the cDNA sequence.^21^This approach has been used for a wide variety of primary immunodeficiencies with promising results.^22^ However, this process of editing involves the induction of a double-strand break and viral transduction in a DNA damage-sensitive stem cell compartment, and consequently impacts the viability, proliferation, engraftment ability, and genomic integrity of the HSPCs.^23–25^ It has been observed that repopulation in mouse models is typically sporadic and oligoclonal^26^, indicating that relatively few long-term repopulating HSPCs are successfully edited without loss of *in vivo* function. *In vitro* optimisation of editing conditions prior to *in vivo* experiments can be time-consuming and recursive, and the unclear link between *in vitro* and *in vivo* makes setting clear performance goals or endpoints challenging. We sought to minimise the toxic effects of the gene editing protocol through a formalised *in vitro* optimisation strategy that determines the best trade-off between correction and functionality of HSPCs in a single definitive experiment. We considered that a trade-off must occur between the percentage of corrected cells, the total number of viable cells, and the ability of those cells to engraft. The first two are readily assessed *in vitro. In vitro* predictors of *in vivo* engraftment are not well established in the context of editing, but for human cord blood transplants engraftment is best predicted by the number of progenitor cell colony-forming units (CFU) in a methylcellulose assay, rather than CD34+ or total viable cell counts.^27^

Response Surface Methodology (RSM) is a well-established family of techniques used extensively for process optimisation in the manufacturing industries which allows modelling of multiple responses in relation to input factors.^28^ Using these techniques, it is possible to generate an accurate model without assessing every possible combination of input parameters. Instead, limited combinations of different levels of each factor are used in a Central Composite Design and a regression model is fitted to each output.^29^ Combinations can be assessed consecutively or concurrently. The fit and predictive robustness of the model can be statistically assessed, and it is then possible to predict the output value for given inputs, or, conversely, to generate predicted input values that maximise or minimise one or multiple output values simultaneously. It is therefore possible to definitively find an optimum set of conditions in a single experimental run, without requiring testing of all possible combinations or needing to reiterate an experiment with altered conditions, a necessary and time-consuming feature of optimising one factor at a time.

We therefore have used a Central Composite RSM design to simultaneously optimise the quantities of Cas9, guide RNA, and AAV-based donor template *in vitro* with respect to frequency of cDNA knock-in, the number of viable cells post-editing, and Colony-Forming Unit (CFU) capacity in semi-solid culture. The models were robustly predictive and revealed an optimum set of conditions that was, unexpectedly, consistent across different CD34+ donors.

When using the optimal protocol in CD34+ HPSCs from a p67^phox^-deficient CGD patient, we achieved the predicted frequency of gene insertion and the rescue of NADPH oxidase activity to normal levels with a considerable reduction of AAV.

## Results

### Gene editing rescues normal levels of p67^phox^ protein in a cellular model of p67-CGD

**Figure 1:**
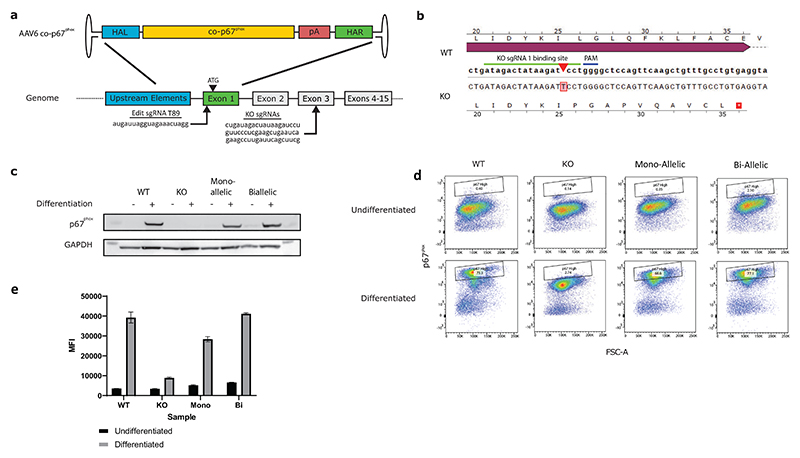
Development of a gene editing platform for the treatment of p67-CGD. **a)** The AAV6 co-p67^phox^, used as an HDR donor, comprises 400 bp left (HAL) and right (HAR) homology arms, a codon-optimised p67 ^phox^ cDNA exon 1-15 (co-p67 ^phox^) and a BGH poly-A sequence (pA). A representation of the *NCF2* locus is shown, including the sgRNA sequence (sgRNAT89) cutting in exon 1 (15 nucleotides before the ATG), and the 3 sgRNAs used to knock out the genomic *NCF2* by mutating exon 3. Arrows show the approximate cut site of sgRNAs. **b)** Sequence of wildtype and knockout NCF2 Exon 3. Sequencing confirmed an inactivating frameshift mutation in Exon 3, in which a T-insertion leads to a premature stop codon. **c-d)** Expression of p67 ^phox^ by wildtype PLB-985s, a knockout clone (KO) and mono and bi-allelic corrected clones, with and without differentiation into neutrophils as assessed by **(c)** Western blot and **(d)** Flow cytometry, with Median Fluorescence Intensities of three technical replicates (±SD) shown for each condition **(e)**.

To mediate the insertion of a therapeutic codon-optimised p67^phox^ cDNA in its own genomic locus we designed a Cas9 ribonucleoprotein complex (RNP) with a single guide RNA (sgRNA) targeting exon 1 immediately before the start codon of the native *NCF2* gene. As a donor template for HDR we created a recombinant AAV2/6 vector containing a codon-optimised p67^phox^ cDNA (exons 1-15) followed by the bovine growth hormone (BGH) poly-A sequence, flanked on either side by 400 bp regions of homology with the DNA sequences surrounding the Cas9 target site, here referred as AAV6 co-p67^phox^ (Figure 1a). To test the new gene editing tools, we created a myeloid cell line deficient for the p67^phox^ protein by targeting *NCF2* exon 3 with three different sgRNA:Cas9 complexes simultaneously in PLB985 cells. Cells were then single cell diluted and expanded into clonal populations, with the knockout validated by sequencing (Figure 1b) and western blot (Figure 1c). The knockout cell line was then corrected by electroporation with a Cas9 + sgRNA T89 RNP followed by transduction with the AAV6 co-p67^phox^ construct at a multiplicity of infection (MOI) of 20,000. Clonal cell lines were again derived from these cells and mono-allelic and bi-allelic corrected clones identified (Figure S1). These clones expressed p67^phox^ at levels close to wildtype only when differentiated (Figure1c, d, e), demonstrating that the donor construct contained all the necessary elements for physiological expression.

### Response Surface Methodology (RSM) can be used to find optimum gene editing parameters in a single experiment

Having validated the functionality of the correction strategy in a cell line, we needed to develop and optimise its use in primary CD34+ cells. As discussed, optimisation of gene correction for HSPCs requires balancing the resulting proportion of corrected cells with the growth inhibition and loss of function that can result from editing.

**Figure 2:**
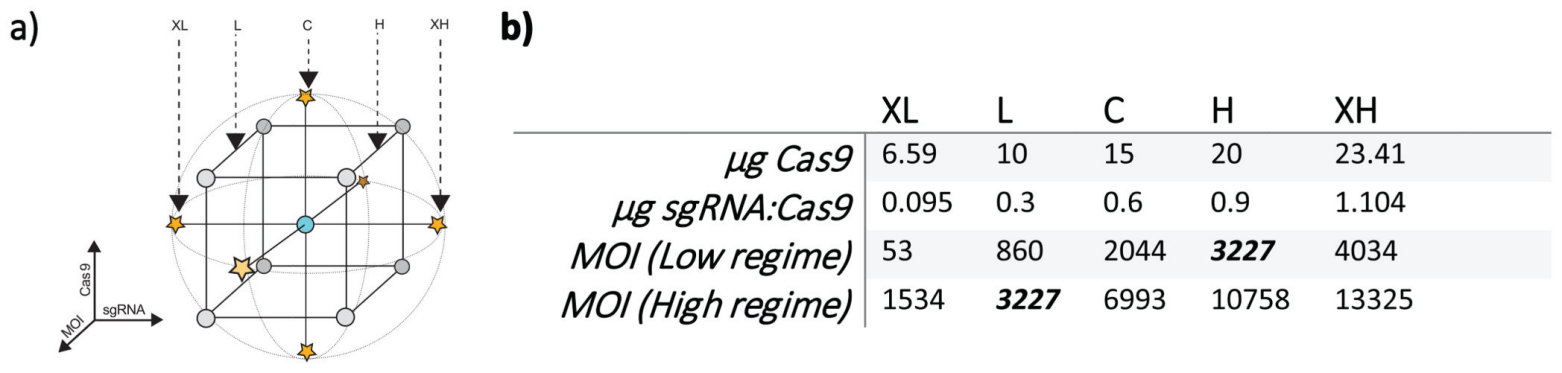
Design of the RSM experiment showing values selected for each level of each factor. The experiment follows a central composite design (CCD) (a). Levels are tested in three types of combinations: all central points, Low/High points (“Cube” points), and Central/Extreme points (“Star” points). The left panel shows a RSM design for three factors. 17 combinations are tested in this design: 3 repeated centre points (CCC), 8 “Cube” points (L/L/L, L/L/H, L/H/L, H/L/L, H/H/L, H/L/H, L/H/H, H/H/H), and 6 “Star” points (C/C/XL, C/C/XH, C/XL/C, C/XH/C, XL/C/C, XH/C/C,). Values are chosen such that these combinations are rotatable with respect to the central set of conditions. Two overlapping CCDs are used (b), with the Low MOI conditions for the first design identical to the High MOI conditions for the second (shown in bold italics), giving 9 MOI levels in total. With the overlapping design, 30 combinations are tested (17 + 17 – 4 overlapping MOI “Cube” point conditions).

To maximise gene correction in HSPC minimising the loss of viable cells and colony-forming units we focussed on the concentration of Cas9, mass ratio of sgRNA to Cas9, and AAV MOI. Rather than varying one factor at a time and selecting one of a few pre-selected concentrations, we used a multidimensional response surface approach.^29^ A response surface can be considered to be a regression line describing the values of a dependent variable, extended into additional dimensions when there is more than one independent variable (here, the independent variables are Cas9, sgRNA:Cas9, and AAV MOI). The central composite design (Figure 2) is a general-purpose parsimonious experimental design in which datapoints are collected for five levels (here referred to as extreme low (XL), low (L), central (C), high (H) and extreme high (XH) for each independent factor in three different categories of combination. The central point (“centre”) consists of the central level for every factor, and is repeated, contributing to estimation of random error. The corner (or “cube”) points consist of every combination of low (L) and high (H) values, and the axial (or “star”) points every combination of one extreme value and all other central values. The experimenter chooses the values of the central and corner points, with the values of the axial points automatically defined such that the design is “rotatable”^30^, meaning that the higher-dimensional “distance” between the central and other points is the same, ensuring equal variance of prediction in all directions. For a design with three factors, this reduces the number of possible five-level combinations from 125 (5^3^) to 17 (Figure 2, left panel). In initial testing we found that addition data resolution was required for low MOI values, and so we combined two overlapping designs for high and low MOI values, with 9 possible MOI levels. The number of testing conditions was thereby reduced from 225 (5^2^x9) to 30 (Figure 2, right panel). Overall, this approach allows for simultaneous covariation of factors to uncover any interactions, allows interpolation between data points to find an exact optimum, and gives an overview of the response “landscape”.

**Figure 3:**
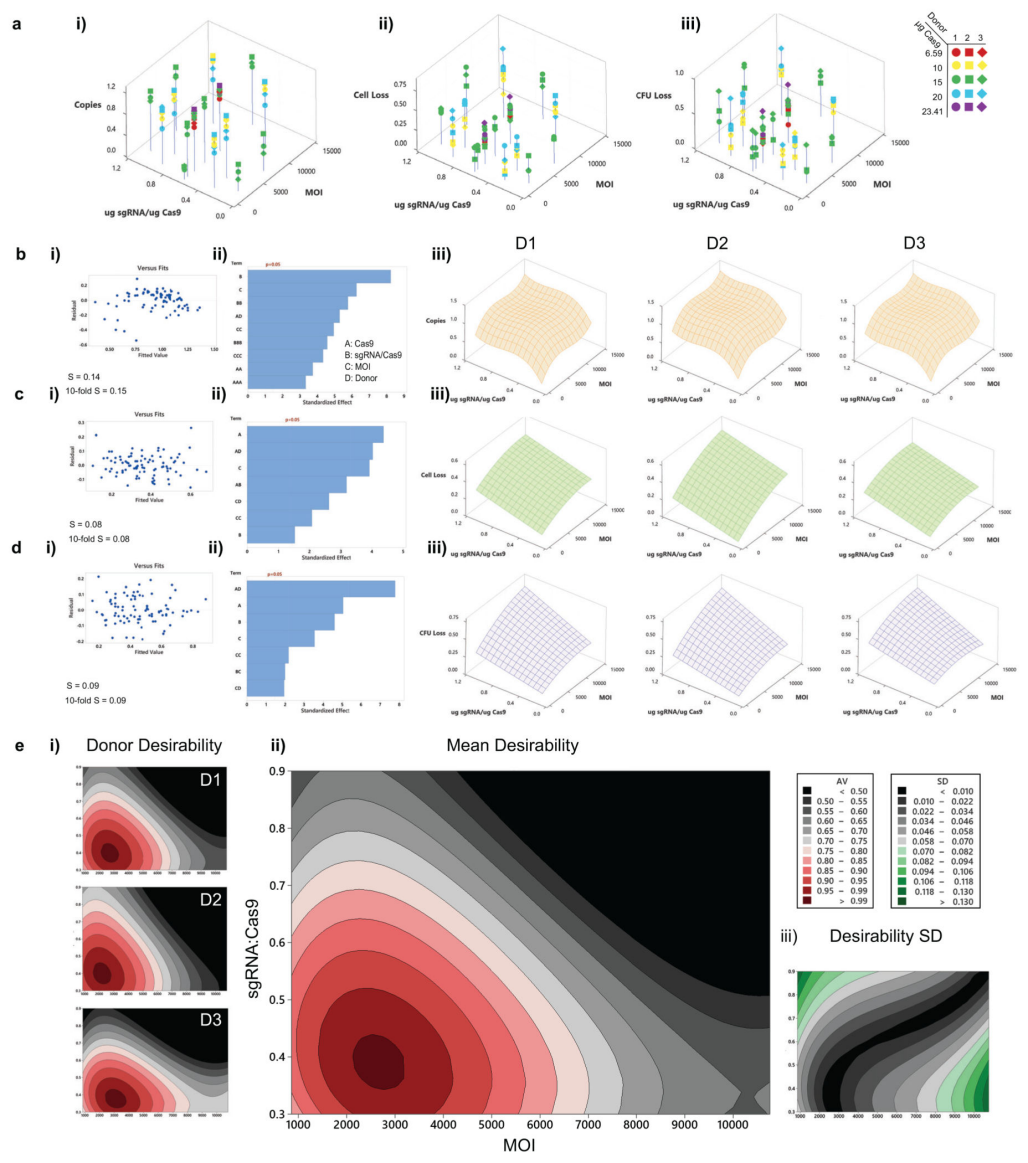
RSM for the optimization of parameters for the gene editing of p67-CGD **a)** The raw data for all three replicates is shown for i) fractional copy number, ii) the fractional reduction in total cell number at Day 5 compared to an electroporation-only control and iii) the fractional reduction in colony-forming units compared to an electroporation-only control. **b)** The fitting of the regression model of fractional copy number. i) shows residuals vs fits indicating good fit except at the lowest observed copy number values. S is a measure of fit given by the average distance of an observed data point from the models’ prediction of that point. 10-fold S indicates the predictive power of the model, with a value close to S indicating good predictive capacity. R^2^ cannot be used as an indicator of fit as the intercept for the model is set to 0. ii) The terms included in the model. Terms with an α < 0.15 were included in the stepwise model generation. Terms below α = 0.05 (p < 0.05) are statistically significant and are ranked by standardised effect size. Higher-order and interacting terms are included as described in the methods. iii) The response surface of the model for copy number for Donors 1-3 (Cas9 kept constant at 8 μg/10^6^ cells). **c)** and **(d)** are as for **(b)** but for Cell loss and CFU Loss respectively. **e)** Desirability functions for maximisation of copy number and minimisation of Cell and CFU loss are shown for i) each donor/replicate and ii) as a normalised average of the 3 donor/replicates with iii) standard deviation.

CD34+ HSPCs were recovered for two days post-thaw, before electroporation and addition to AAV-containing media as described in methods. After overnight transduction, CD34+ were split, plated in semisolid media for 14-16 days before quantification of colony-forming units (CFU), and separately maintained in liquid culture until Day 5 post-editing, at which point cells were counted to assess cell number and viability and then harvested to determine the average number of integrated cDNAs by droplet digital PCR (ddPCR). This value is expressed as a fraction (integrations per diploid genome) and referred to as fractional copy number. Figure 3a shows i) fractional copy number and decline in ii) total live cells and iii) CFUs compared to mock-edited (WT) HSPCs (n=3 different donors). As *NCF2* is autosomal, up to two copies of the construct may theoretically be integrated by HDR per cell.

Electroporation alone (WT-E) resulted in a 14 ± 8 % loss of cell number and a 13 ± 6 % loss of CFUs (Figure S2). A multidimensional polynomial regression was carried out for each response with Cas9 concentration, sgRNA: Cas9 ratio and AAV MOI as continuous variables and HPSC donor/Experimental replicate as a categorical blocking variable, using a stepwise approach for inclusion of terms as described in the methods. The models were generally well-fitted {Figure 3b) i), c) i), d) i)}, although the differences between predicted and observed values (residuals) for copy number indicated less effective fitting at the lowest fractional copy number values. Model predictivity was assessed by k (10)-fold cross-validation; near-equivalence of standard and 10-fold S values indicated that the models were robustly predictive.

Differences between donor/replicate sensitivity to Cas9 concentration, sgRNA: Cas9 ratio and AAV MOI were apparent by the significance of interacting terms (AD, BD, CD) {Figure 3b) ii), c) ii), d) ii)}, with donor/replicate-specific sensitivity to Cas9 concentration being a major contributor to loss of cells and CFUs. Plotting the regression models in 3D shows a clear trend across HSPC donor/replicates. The fractional copy number initially increased with increasing AAV MOI and sgRNA:Cas9 ratio before rapidly plateauing (Figure 3b)iii)). Contrastingly, with increasing MOI, sgRNA:Cas9 ratio, and Cas9, the total number of cells (c)iii)) and CFUs (d)iii)) declined continuously compared to the mock-electroporated control. Increasing Cas9 appeared to slightly reduce copy number, implying that sgRNA was limiting for nuclease activity and that Cas9 had a deleterious effect independent of nuclease activity.

Because of the diminishing returns for fractional copy number and continuous losses for total viable cells and CFUs when increasing Cas9, sgRNA:Cas9 ratio and AAV MOI, an optimum set of conditions necessarily exists. We carried out a regression optimisation, giving equal weight and importance to each response to calculate a desirability function for each replicate/donor (Figure 3e) i), details of which are given in the methods. Response optimisation was carried out within the well-fitted region between the upper and lower bounds of each variable. The optimisation process generates a nominal optimum set of conditions which maximises the desirability function for each donor/replicate. Showing the overall desirability landscape reveals changes that can be expected when deviating from these values through choice or random error.

Despite the variability in the sensitivity to editing and resulting copy numbers for each donor/replicate, the desirability functions for each HSPC donor source were remarkably similar. An average normalised cross-donor desirability plot is shown in Figure 3e) ii). The optimum for Cas9 concentration was found to be at or below the lower bound for fitting (10 μg) for all donors. For MOI and sgRNA: Cas9 ratio variables, broad, overlapping peaks of >99% normalised desirability between approximately MOI 1000-4000 and sgRNA:Cas9 0.35-0.45 were found for all donor/replicates, and standard deviation between predicted desirability between donor/replicates was lowest in this optimum region specifically (Figure 3e) iii). A set of conditions at the centre of the 99^th^ percentile of mean desirability (10 μg Cas9, sgRNA:Cas9 ratio 0.4, MOI 2800) is within the 99^th^ percentile of desirability for each individual donor/replicate. Beyond these broad peaks, the small increases in copy number achieved by using a higher MOI or sgRNA:Cas9 ratio were outweighed by the concomitant loss of cells and CFUs.

**Figure 4:**
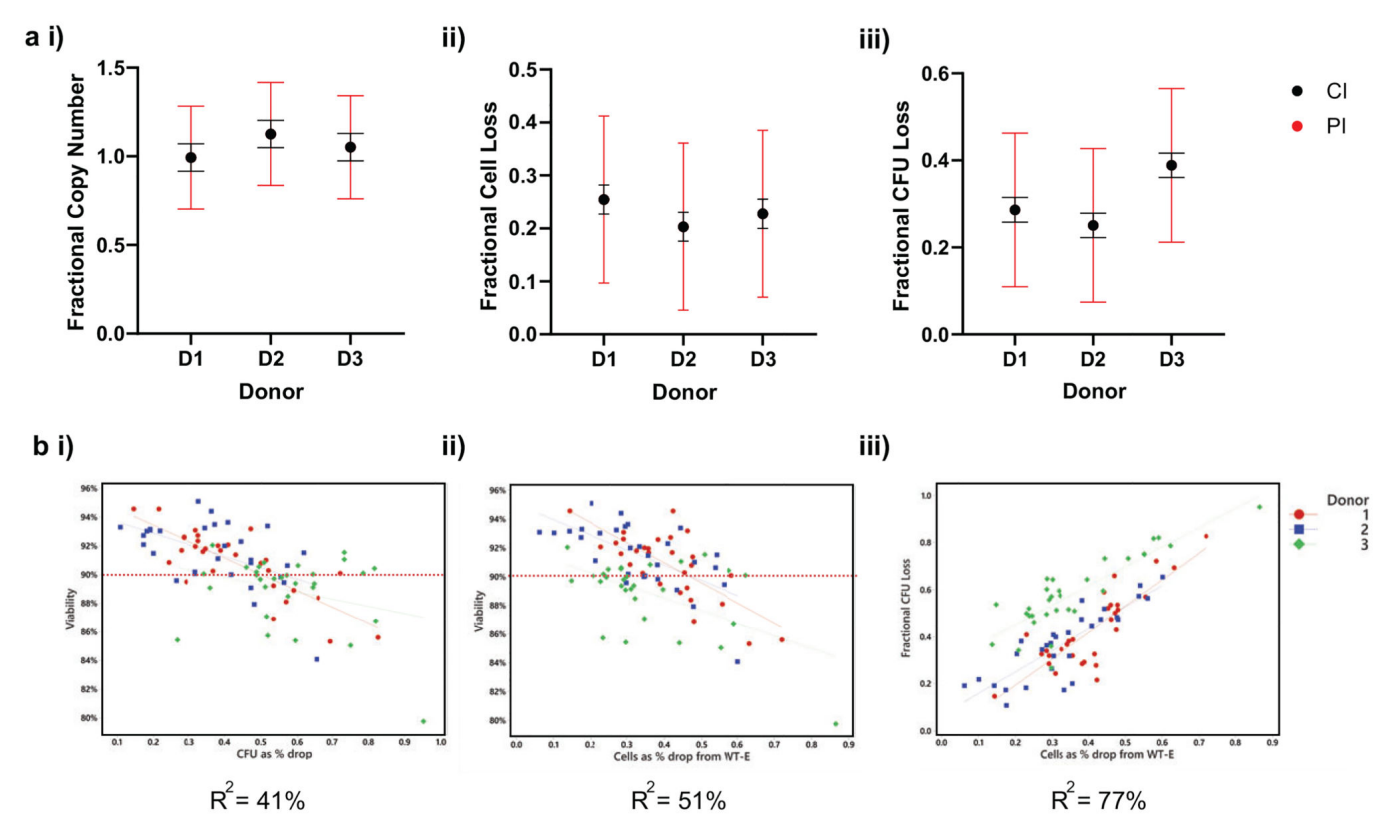
Predictions and relationship to viability. **a)** Predicted mean values for each donor/replicate are shown with error bars indicating confidence interval (95% confident true mean within this range) and predictive interval (95% confident new observations will fall in this range), for Copy number (i), Fractional cell loss (ii) and Fractional CFU loss (iii). **b)** Correlations between i) Viability and CFU loss, ii) Viability and Cell Loss, and iii) CFU loss and Cell loss are shown.

The 95% confidence (CI) and predictive intervals (PI) can be calculated for the estimated outputs when using this set of conditions, where the confidence interval is the likely range of the true mean value, and the predictive interval is the likely range of new observations. The predicted mean and 95% predictive intervals for copy number (i), fractional cell loss (ii), and fractional CFU loss (iii) for 2800 MOI, 0.4 sgRNA:Cas9 ratio, and 10 μg Cas9 are shown in Figure 4 a). There were some incidental findings resulting from the overview of the response landscape. Although the percentage of viable cells is frequently used as a metric of fitness, viability did not correlate strongly with total cell number or total CFU (Figure 4b).

Interestingly, we found commonalities between unrelated gene editing constructs. When the RSM was applied to a gene editing platform targeting the *WAS* gene for Wiskott-Aldrich Syndrome^31^ we found that Cas9 concentration, sgRNA:Cas9 ratio, and MOI influenced the outcome of editing in much the same way as observed for integration of codon-optimised p67^phox^ cDNA at the *NCF2* locus in Figure 3, with a similar plateau of editing efficiency alongside increasing losses of total cell number and CFUs (Figures S3 and S4). Notably, the optimum conditions were also very similar even though a completely different AAV vector and sgRNA were used, with a 99% desirability optimum at 10 μg Cas9 and a sgRNA:Cas9 mass ratio between 0.35-0.5. The 99% optimum MOI range was approximately half that of AAV6 co-p67^phox^, between 1000-3000.

**Figure 5:**
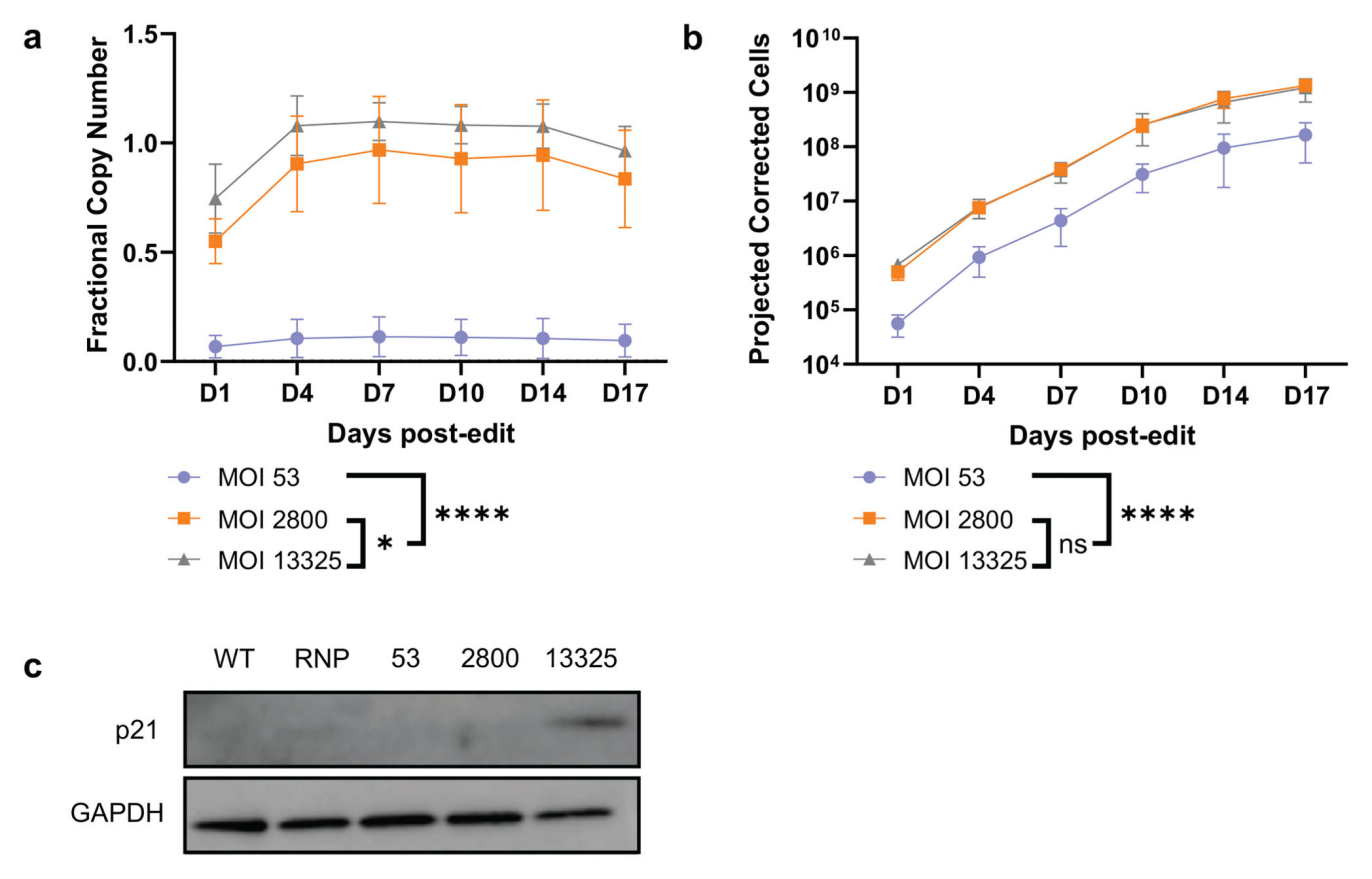
Testing the optimality of AAV6 co-p67^phox^ at MOI 2800. HSPCs were edited as before with MOIs of 53 (low), 2800 (optimum), and 13325 (high). Copy number over time is shown in panel **a)** and the projected number of corrected cells at each timepoint in panel **b)** (n=3; mean ± SD. *: p<0.05, ****: p<0.0001 (2-way ANOVA & Post-Hoc Tukey test). **c)** Western blotting showing expression of p21 in response to editing at Day 1 (n=2).

To validate our prediction that an MOI of 2800 would maximise the number of functional p67-corrected cells, we edited CD34+ cells from three new donors with a range of MOIs (low; MOI 53, optimal; MOI 2800, high; MOI 13325), and initiated neutrophil differentiation to investigate whether there were differences between MOIs in survival of edited cells over a longer-term expansion. At each timepoint, cell concentration was re-normalised to 10^6^/ml. Copy number stabilised by Day 4 and remained stable throughout for all conditions (Figure 5a). We estimated the number of corrected cells by multiplying the number of viable cells by fractional copy number at each timepoint (Figure 5b). Increasing MOI led to statistically significant increases in copy number between conditions. When cell number was considered to estimate the total number of corrected cells, there was no significant difference between MOI 2800 and 13325, emphasizing once more the adequacy of using reduced doses of AAV to achieve correction. p53 activation is known to be involved in the response to AAV in a gene editing context.^25^ We therefore additionally assessed expression of a target of p53, the DNA damage response downstream effector CDKN1A (here referred to as p21). We detected expression of p21 in the MOI 13325 condition only, with no apparent expression in the MOI 2800 or other conditions (Figure 5b). We concluded that there was no disadvantage to a substantial reduction in AAV MOI used for correction, if used at or around the optimum value, with a potential advantage in reduction of damage response signalling.

### Rescue of the CGD phenotype by gene editing

**Figure 6:**
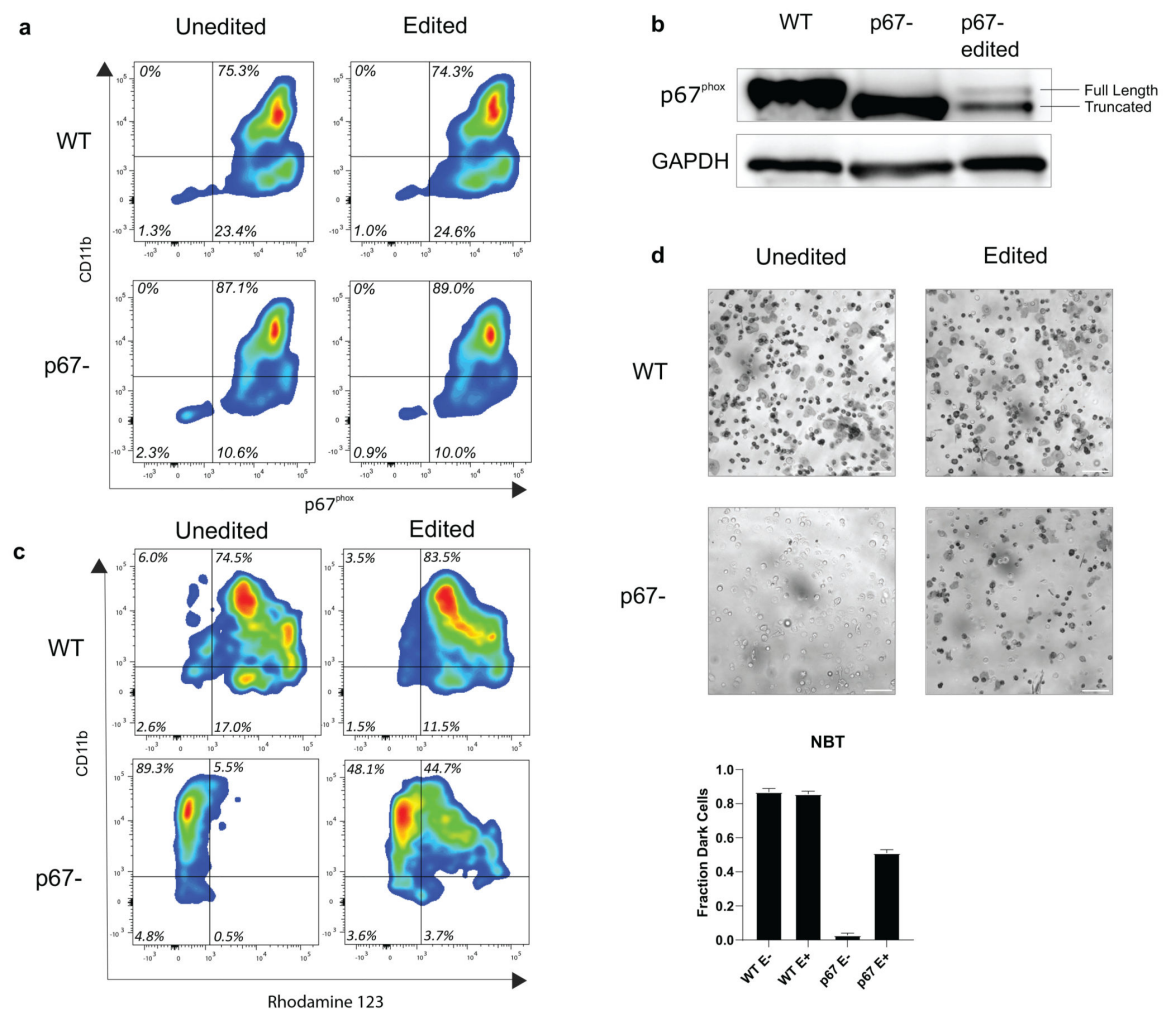
Functional correction of patient-derived p67^phox^-deficient cells. **a-b)** Neutrophil differentiation (as assessed by cd11b expression) and p67^phox^ expression of wildtype (WT) and p67^phox^-deficient (p67-) primary CD34+ cells by flow cytometry **(a)** and western blotting **(b). c)** Dihydrorhodamine (DHR) assay (shown is the percentage of rhodamine 123 +ve out of cd11b +ve cells) and **d)** Nitro Blue Tetrazolium (NBT) assay of WT, patient cells and gene edited cells. Scale bar = 50 μm.

We tested the ability of our optimised protocol to correct CD34+ cells from a patient with p67^phox^ -deficient CGD (Bakri et al^5^, patient M2). Unmobilised peripheral bloods from the patient and a healthy volunteer were obtained under informed consent, and CD34+ cells extracted by magnetic immunoaffinity selection. Only 4 mL of blood could be collected from the patient and so pre-editing expansion was extended from 2 to 7 days. After 7 days, approximately 400,000 patient-derived cells were obtained, with over 90% of cells CD34+. 300,000 cells were edited with the remainder kept as a negative control. At Day 5 post-editing, 60,000 cells were taken for neutrophil differentiation. Remaining CD34+ cells were expanded in maintenance media for an additional 7 days prior to genomic DNA extraction. The fractional copy number of the edited patient cells at this point was 1.01 ± 0.02. At day 16 of differentiation, cells were analysed by flow cytometry and found to express cd11b (indicating myeloid differentiation) and p67^phox^ (Figure 6a). Surprisingly, the uncorrected patient cells also expressed levels of p67^phox^ comparable to the wildtype control. We hypothesised that as the patient carries an exon 6 deletion that disrupts the activation domain of p67^phox^ but does not result in a frameshift^5^, a truncated form of the protein was still being expressed. We observed this truncated form by Western blot (Figure 6b) and observed that the corrected patient cells expressed both the truncated and full-length form (at reduced levels compared to wildtype and unedited patient cells). The reduced levels of the truncated protein could be easily explained by the “knock in” of the donor template and to some extent by potentially inactivating indels (Figure S6). The lower expression of the wildtype protein is probably due to a prevalence of monoallelic over biallelic correction or possibly to the lack of intronic regulatory elements in the donor template. The latter seems unlikely considering the physiological levels of expression seen in corrected clonal cell lines (Figure 1). An overall reduction of protein may also reflect minor differences in the differentiation stage of the samples. More importantly, the levels of NADPH oxidase expression support function as shown below. Differentiated cells were assessed for their ability to generate ROS in response to Phorbol-12-myristate-13-Acetate (PMA). Dihydrorhodamine (DHR) (Figure 6c) and Nitroblue Tetrazolium NBT (Figure 6d) assays for generation of ROS in response to stimulus confirmed that patient cells were unable to generate ROS and were rescued by our gene editing protocol. Across both assays, approximately half of the corrected cells produced ROS to a comparable level of wildtype, compared to 85-95 % of wildtype cells, indicating robust functional correction per cell. The copy number of the differentiated cells was 0.83 ± 0.02, indicating a small loss of edited cells during differentiation, and a mixture of mono and bi-allelic correction.

## Discussion

In this study we developed, optimised, and validated a CRISPR/Cas9 AAV HDR strategy for the correction of p67^phox^-deficient CGD, by insertion of an intron-less codon-optimise p67^phox^ cDNA at the native genomic *NCF2* locus. We used an RSM-based approach to discover an optimum set of conditions for the *in vitro* edit, balancing the percentage of corrected cells with the loss of cells and colony-forming units that occurs as a result of the edit. We found a general pattern across HSPC donor sources and constructs in which there were rapidly diminishing returns for increased sgRNA and AAV in terms of percentage correction, but with continuous losses of cells and CFU, leading to a straightforward optimisation rationale in which the best conditions for editing are those just before the correction plateau.

RSM allowed us to progress through the initial optimisation stages of development rapidly, with each experiment requiring a few hours of hands-on work on 5 days over a 2-week period. This approach can in principle be used for any continuous variable and could be extended to aspects of construct design (e.g. length of homology arms) or the electroporation itself (e.g. voltage, pulse length). Given the initial rise and rapid plateauing, the relationship between fractional copy number and AAV MOI and sgRNA:Cas9 ratio is likely more properly modelled by a logarithmic model than a polynomial; however, not using the same model equation for each output could bias the conclusions of the analysis by implicitly assuming a plateau in copy number and not in the other outputs. Therefore, we judged that using a polynomial model for all outputs was most appropriate.

Interestingly, we observed that viability after editing did not correlate well with cell fitness. In some conditions, cells were measured to be over 90% viable but had over a 60% reduction in CFU and cell number. Contrastingly, the reductions in CFU and total cell number correlated well. As CFU and cell number losses are correlated and may reflect the same underlying biological events, optimising with respect to both might have led to over-weighting of deleterious effects in the optimisation. However, optimisation with respect to copy number and cells alone or copy number and CFU alone still places the previously identified conditions within the 90^th^ and 95^th^ percentile of optimality respectively (Figure S5). An unexpected observation was that the optimum was consistent across HSPC donor sources for both the p67-CGD and WAS platforms. Part of our rationale for developing the RSM approach was to address the fact that different CD34+ donors are known to vary in their editing frequency, viability, and repopulation ability. We considered that a quick and defined process with a clear endpoint might be advantageous if it were found to be beneficial to determine donor-specific optimum conditions prior to treatment. Unexpectedly, we found that despite donor-to-donor variation of editing rates, sensitivity to AAV and nuclease activity being evident within our experiments, the trade-off between these factors occurred at approximately the same set of conditions across donor/replicates and so optimisation for each new HSPC donor would not be beneficial. As each replicate was an independent HSPC donor, differences between replicates cannot be attributed solely to biological properties of the donors as random effects will also contribute. We interpret this to mean that it is likely sufficient to carry out optimisation for each new AAV/CRISPR combination only and not per-donor. Intriguingly, the average predicted copy number at the optimum was ~1 for p67-CGD (Figure 4a) and ~0.5 for WAS for all donors tested (Figure S4). As p67-CGD is autosomal and WAS is X-linked, in both cases this represent 50% correction. Speculatively, it may be possible that 50% correction is a general optimum. If the addition of more AAV or nuclease results in a fitness cost independent of correction, at above 50% population correction it is more likely than not that any additional AAV/nuclease entering a random cell will add a fitness cost without increasing the number of corrected cells, as half of cells will already be corrected. Additional editing might therefore lower the overall corrected fitness of the population. However, we have not explicitly demonstrated that this is the case, and when calculating optima based only on copy number vs cell number or copy number vs CFU, the optimum copy number was higher (Figure S5). Other factors, such as differences between vectors or AAV preparations (e.g. capsid empty/full ratios and purity) may also have contributed to the observed difference in optimum MOIs.

The RSM suggests that achieving optimal gene editing results does not require the use of high doses of AAV (MOI 13325) and such high doses may even have negative consequences. We found that overall, the optimal dose of AAV for the gene editing of p67-CGD (MOI 2800) and WAS (MOI 1400) is substantially lower than those used in many other reports.^11,32–34^ Besides improving the number and quality of the cells available for transplant (the use of MOI 2800 does not result in p21 activation that could impact cell function), this is also advantageous for reducing AAV manufacturing costs for clinical applications.^35^

In the last part of the study, the optimal conditions were applied to CD34+ cells from a p67^phox^-deficient CGD patient. Patient-derived CD34+ cells were obtained from 4 mL of unmobilised peripheral blood by positive bead selection followed by an extended pre-correction culture. Despite the extended culture, cells retained expression of the CD34+ marker and retained the ability to differentiate. As previously reported, the differentiated patient cells had no detectable ROS activity.^5^ Unexpectedly, the patient cells expressed p67^phox^ as detected by flow cytometry, which was revealed to be a truncated form likely not detected in previous work due to differences in detection antibody specificity. This limited our ability to quantify expression at the protein level; however, we observed functional correction of patient-derived cells. Approximately half of edited patient cells were able to generate ROS as measured by functional assays. Analysis of targeted integration immediately after editing revealed ~1 copy per cell on average, and ~0.8 at the time of analysis after 19 days of differentiation, indicating a mixture of monoallelic and biallelic insertion, as for ~50% functional correction at day 19 the integrated copy number would have to be ~0.5 for fully monoallelic insertion and ~1 for fully biallelic.

Studies on carriers of X-linked CGD report that 20% of functional neutrophils are required to confer protection against life-threatening infections, although lower levels also have very significant positive effects.^6^ Based on the above, the levels of correction we obtain with our gene editing protocol for p67-CGD are likely to confer clinical benefits.

This work demonstrates the potential for a CRISPR/Cas9 HDR gene therapy for correction of p67-CGD in patient-derived CD34+ cells, and the potential to expedite and simplify pre-clinical development of gene therapies in general by adoption of RSM optimisation techniques, which in this case allowed definitive optimisation of three different parameters in a single experiment while reducing considerably (from 225 to 30) the number of required combinations of conditions for optimisation.

## Materials and Methods

### Cell culture

*HPSCs* were isolated from aphereses using Miltenyi MS columns with CD34 binding beads according to manufacturer instructions HPSCs were cultured in StemSpan II supplemented with 100 ng/mL Flt3-Ligand, 100 ng/mL Stem Cell Factor (SCF), 20 ng/mL Thrombopoietin (TPO), 20 ng/mL Interleukin-6 (IL-6), 60 ng/mL Interleukin-3 (IL-3), 1 μM StemRegenin-1 (SR-1), 50 nM UM171, and 1% Penicillin/Streptomycin (P/S). Cytokines were purchased from Peprotech (London, UK), Stemspan II, SR-1 and UM171 from STEMCELL Tech (Vancouver, Canada), and Pen/Strep from Gibco (MA, USA). *CD34+ isolation and expansion from peripheral blood*. Peripheral Blood Mononuclear Cells (PBMCs) were isolated from unmobilised peripheral blood by 3-fold dilution in PBS followed by Ficoll-Paque density gradient centrifugation (25 min, 400 g, brakes off). Isolated PBMCs were diluted in PBS, passed through a 30 μm cell strainer, pelleted (10 min, 300 g) and resuspended in MACS buffer (PBS + 1% BSA). CD34+ cells were isolated with Miltenyi MS columns and CD34+ selection beads according to manufacturer instructions. Eluted cells were resuspended without counting in 500 μL of StemSpan II with supplements as above for 5 days. On Day 4, an additional 1.5 mL of fresh media was added with double amount of Stem Regenin. On Day 7, cells were counted, analysed by FACS for CD34+ expression, and edited as described below. *PLB985* were grown in suspension in RPMI 1640 + 10% Fetal Bovine Serum (FBS) + 1% P/S (Gibco). *HEK-293T* were grown as an adherent culture in DMEM + 10% Fetal Bovine Serum (FBS) + 1% P/S (Gibco). Cells were counted manually using a 1:1 dilution of Trypan Blue (Gibco) and disposable Neubauer C-Chips (NanoEnTek, South Korea). For each sample, 2 chambers were loaded, and four quadrant counts taken per chamber. When counting Day 5 post-electroporation, cells were not pelleted to avoid variable resuspension losses; instead, cells were resuspended in their media and the volume of media measured.

### Molecular Cloning and AAV6 preparation

All cloning was carried out using the NEBuilder HiFi assembly Cloning Kit (New England Biolabs, MA, USA). Codon-optimised cDNA sequences were synthesised by Invitrogen Geneart. Constructs were transformed into either DH5α (NEB) or Stbl3 (ThermoFisher, MA, USA) competent cells and grown in Luria Broth (LB). Plasmid isolation was carried out using either the Monarch Plasmid Miniprep Kit (NEB) or the Qiagen Plasmid Maxi Kit (Qiagen, Hilden, Germany). Plasmid sequences were verified by sequencing (Eurofins, Luxembourg). The p67^phox^ transfer Plasmid consists of an AAV2 backbone derived from pAAV-CAG-GFP, which was a gift from Edward Boyden (Addgene plasmid #37825), the upstream 400 bp homology arm, the codon-optimised *NCF-2* coding sequence, bovine growth hormone poly-adenylation signal, and the downstream 400 bp homology arm. The WAS transfer plasmid has the same components but with a co WAS sequence and WAS-targeted homology arms. AAV6 were produced by transient transfection on HEK293T cells of the transfer and the pDGM6 Rep/Cap helper plasmids (as described in Supplementary materials).

### PLB-985 Editing and Single Cell Cloning

To generate knockout cells, PLB-985 cells were electroporated as for HSPCs, using 3 pooled sgRNAs targeting *NCF2* exon 3, using the “Optimization 3” pulse setting on a Maxcyte GTx Gen2 Electroporator and RPMI + 10% FBS media. Cells were allowed to expand for 3 days before diluting in RPMI + 20% FBS + P/S to a concentration of 500.000 cells/ml. 100 μLwas added to each well of 20x U-bottom 96-well plates. After 3 weeks, single cell-colonies were resuspended and consolidated into 1 plate. Genomic DNA was extracted, the knockout locus amplified and sequenced. Biallelic knockouts were identified from ICE analysis (Synthego) of sequence data. Knock-ins were generated the same way, using the T89 sgRNA and AAV6 co-p67^phox^ at an MOI of 25000. Knock-ins were identified by screening using the NBT assay and confirmed by ddPCR.

### Gene editing and RSM optimisation

48 hours post-thaw, HSPCs were pelleted by centrifugation at 300 g, 10 min, resuspended in 5 mL Electroporation Buffer (MaxCyte), counted, pelleted again and resuspended in Electroporation Buffer to a final concentration of 8.33 x10^7^ cells/mL. 1.25 x10^6^ cells were used per condition, except for the editing of patient-derived p67^phox^-negative cells, for which 300,000 cells were used. RNPs were prepared by mixing SpyFi Cas9 (IDT) and T89 sgRNA (Synthego) in the quantities specified in Table S1 for the RSM Optimisation, and 10 μg Cas9:4 μg sgRNA when using the optimised conditions. RNPs were allowed to assemble for 15’ at 37 °C before addition of MaxCyte buffer and resuspended cells to a final volume of 25 μL. 25 μL of the cell/RNP mix was transferred to the chambers of OC-3x25 Electroporation Cuvettes (MaxCyte), ensuring no bubbles within the chambers. Cells were electroporated with a MaxCyte Gtx Gen2, using the “HSC-3” programme. Chambers were returned to the 37 °C incubator for 15’ before recovery and washing with 25 μl of Stemspan. 12 μL (300,000 cells) of electroporated cells were then added to wells of a flat-bottomed 96-well plate containing 188 μl AAV pre-diluted in Stemspan to the appropriate MOIs. AAV transduction was allowed to occur over 16 h before transfer of samples to a V-bottomed 96-well plate, pelleting as before, and resuspension in 1200 μl of fresh Stemspan in 24-well plates.

### Colony-forming Unit assay

For the RSM Optimisation, 10 μL of the resuspended cells were diluted with 490 μL of unsupplemented StemSpan (5000/mL as per pre-edit cell concentration). For patient derived CD34 cells, concentration was 50,000/mL, counted at the time of preparation. 150 μL of diluted cells were added to 1.5 mL aliquots of MethoCult H4435 (StemCell) and vortexed for 5 s. After settling, 1.1 mL was dispensed using a 3 mL Syringe and 18G needle into SmartDish (StemCell) CFU assay plates. These were placed in secondary humidified chambers and incubated at 37 °C for 14-16 days before CFU tallying.

### Myeloid differentiation

PLB-985 cells were differentiated into neutrophils by resuspension at 2.5 x10^5^ cells/mL in Neutrophil Differentiation Media (RPMI 1640 + 0.25% FBS + P/S + 0.5% Di-Methyl Formamide (DMF) + 1x Nutridoma-CS (Sigma-Aldrich). Media was topped up at Day 3 and cells used at Day 5. 60,000 CD34+ cells were resuspended at 300,000 cells/mL in CD34 Neutrophil Differentiation medium (IMDM + 20% FBS, 20 ng/mL IL3, 100 ng/mL G-CSF), with cells counted and resuspended at 300,000 cells/mL every two days for 16 days, prior to analysis of differentiation and expression of p67^phox^.

### Functional studies

#### Dihydrorhodamine (DHR) test

Cells (300,000) were stained with anti CD11b-APC (clone M1/70, Biolegend) before being incubated for 15 min at 37 °C in 1 mL of PBS-gg (0.05% gelatine, 0.09% D-glucose) containing 2.9mM of dihydrorhodamine 123 (DHR; Sigma - Aldrich) and 150U/mL of catalase (Sigma-Aldrich). The samples were then divided into two equal portions (500 mL each) with one receiving 1 mg/mL of PMA (Sigma-Aldrich) and were incubated at 37 °C for further 15 min before being analysed on the LSRII flow cytometer.

#### Nitro Blue Tetrazolium Assay

100 μl of 1 μg/mL Phorbol 12-myristate 13-acetate (PMA) and 1 μg/mL Nitro Blue Tetrazolium (NBT) was added per 96 well. Generation of a blue precipitate reports the presence of reactive oxygen species.

### Flow Cytometry

Cells were pelleted (300 g, 10 min) and resuspended in FACS buffer (PBS + 1% BSA). For live cell FACS of surface markers, cells were resuspended in 50 μL of FACS buffer with 1 μL of each antibody (see Table S2) and incubated on ice in the dark for 30 min. Cells were then resuspended in 1 mL FACS buffer, washed twice, and resuspended in 200 μLFACS buffer before analysis. For analysis of intracellular p67^phox^, cells were fixed and permeabilised using the Nordic-MUbio FIX&PERM kit prior to incubation with anti-p67 antibody (1:50 dilution), incubated for 30’, washed 3x, incubated with Cy5 anti-Rb secondary (30 min), washed 3x, and then kept at 4 °C until analysis.

### ddPCR

Genomic DNA from cell culture was extracted using the Qiagen Blood & Tissue Kit. DNA from blood samples was extracted using the QiaAmp DNA micro kit. Samples were eluted in nuclease-free (NF) water and diluted to 30 ng/uL prior to ddPCR analysis. Reactions were assembled with 11 μL 2x ddPCR Supermix for Probes (no dUTP) (Bio-Rad, CA, USA), 0.8 μL each 10 μM primer, 0.4 μL each 10 μM probe, 2 μL sample DNA, and NF water to 22 μL. Droplets were generated using a QX200 AutoDG, amplified with a C1000 Touch Thermal Cycler, and analysed with a QX200 droplet reader (Bio-Rad). The frequency of integrated copies is reported as fractional copy number (average copies per diploid genome). Primers (Life Tech), probes (IDT) and cycling conditions are shown in Table S3 and S4. AAV ITR detection is adapted from Aurnhammer et al.^36^

### Statistical Analysis

Analyses were performed and graphs generated with Minitab 19 (Response surface analyses) and Graphpad Prism (all other). Response surface regressions were performed using stepwise linear regression with first order interacting terms and up to third-order non-interacting terms, without an intercept term as the intercept was *a priori* zero. The Categorical “Donor” variable was included only as an interacting term. Multiparameter Optimisation was performed using the minitab “Response Optimisation” function, with the boundaries set between the “Low” and “High” values for each variable.

## Supplementary Material

Supplementary Figures

Supplementary Methods
