# Supplementary material for: Multidimensional Response Surface Methodology for the development of a gene editing protocol for p67phox-deficient Chronic Granulomatous Disease
Source: Hum Gene Ther. Author manuscript; Available in PMC 2024 Apr 16. (PMC7615834; doi:10.1089/hum.2023.114)
Supplement: Supplementary Figures [file EMS194183-supplement-Supplementary_Figures.docx]

**Figure S1**

**Figure S1.** **Copy Number Determination in PLB985.** a) ddPCR integration copy number analysis for the mono and bi-allelically integrated clones of the PLB 985 knockout (n = 2 technical replicates). b) Karyotype of i) unmodified and ii) knockout PLB 985 cells.

*Albumin* (our reference gene) is located on chromosome 4q13.3, and *NCF2* at chromosome 1q25.3. In both the wildtype and the knockout line, chromosome 4 is diploid and so the albumin reference is valid. Chromosome 1 exhibits an internal duplication estimated to be between 1q21-23. This does not encompass the *NCF2* locus and so the number of NCF2 sites available for correction are unaffected.

**Figure S2**

**Figure S2. Loss of cell number and CFU due to electroporation alone.** Individual donor data are shown for both p67^phox^ (a) and *WAS* (b) correction, for both total cell number (i) and total CFU number (ii).

**Figure S3**

**Figure S3. Response Surface Optimisation of *WAS* correction.** a) The raw data for all three replicates is shown for i) fractional copy number, ii) the fractional reduction in total cell number at Day 5 compared to an electroporation-only control and iii) the fractional reduction in colony-forming units compared to an electroporation-only control. b) The fitting of the regression model of fractional copy number. b i) shows residuals vs fits indicating good fit except at the lowest observed copy number values. S is a measure of fit given by the average distance of an observed data point from the models’ prediction of that point. 10-fold S indicates the predictive power of the model, with a value close to S indicating good predictive capacity. R^2^ cannot be used as an indicator of fit as the intercept for the model is set to 0. ii) The terms included in the model. Terms with an α < 0.15 were included in the stepwise model generation. Terms below α = 0.05 (p < 0.05) are statistically significant and are ranked by standardised effect size. Higher-order and interacting terms are included as described in the methods. iii) The response surface of the model for copy number for Donors 1-3 (Cas9 kept constant at 10 µg). c and d are as for b but for Cell loss and CFU Loss respectively. e) Desirability functions for maximisation of copy number and minimisation of Cell and CFU loss are shown for i) each donor/replicate and ii) as a normalised average of the 3 donor/replicates with iii) standard deviation.

**Figure S4**

**Figure S4.** **RSM Predictions.** Predictions for a) copy number, b) fractional cell loss and c) fractional CFU loss for the selected set of optimised conditions (MOI 2800, Cas 10 µg, sgRNA/Cas9 0.4). Predictions are shown for each donor. Confidence Interval (CI) represents the estimated range which has a 95% probability of containing the true mean, and Predictive Interval (PI) represents the estimated range which has a 95% probability of containing subsequent observations.

**Figure S5**

**Figure S5. Alternate Desirability Functions.** Desirability functions for maximisation of p67^phox^ copy number and minimisation of a) Cell and b) CFU loss are shown for i) each donor/replicate and ii) as a normalised average of the 3 donor/replicates with iii) standard deviation. The position of the copy number/cfu/cell optimum as described in the main text is shown with a white crosshair.

**Figure S6.**


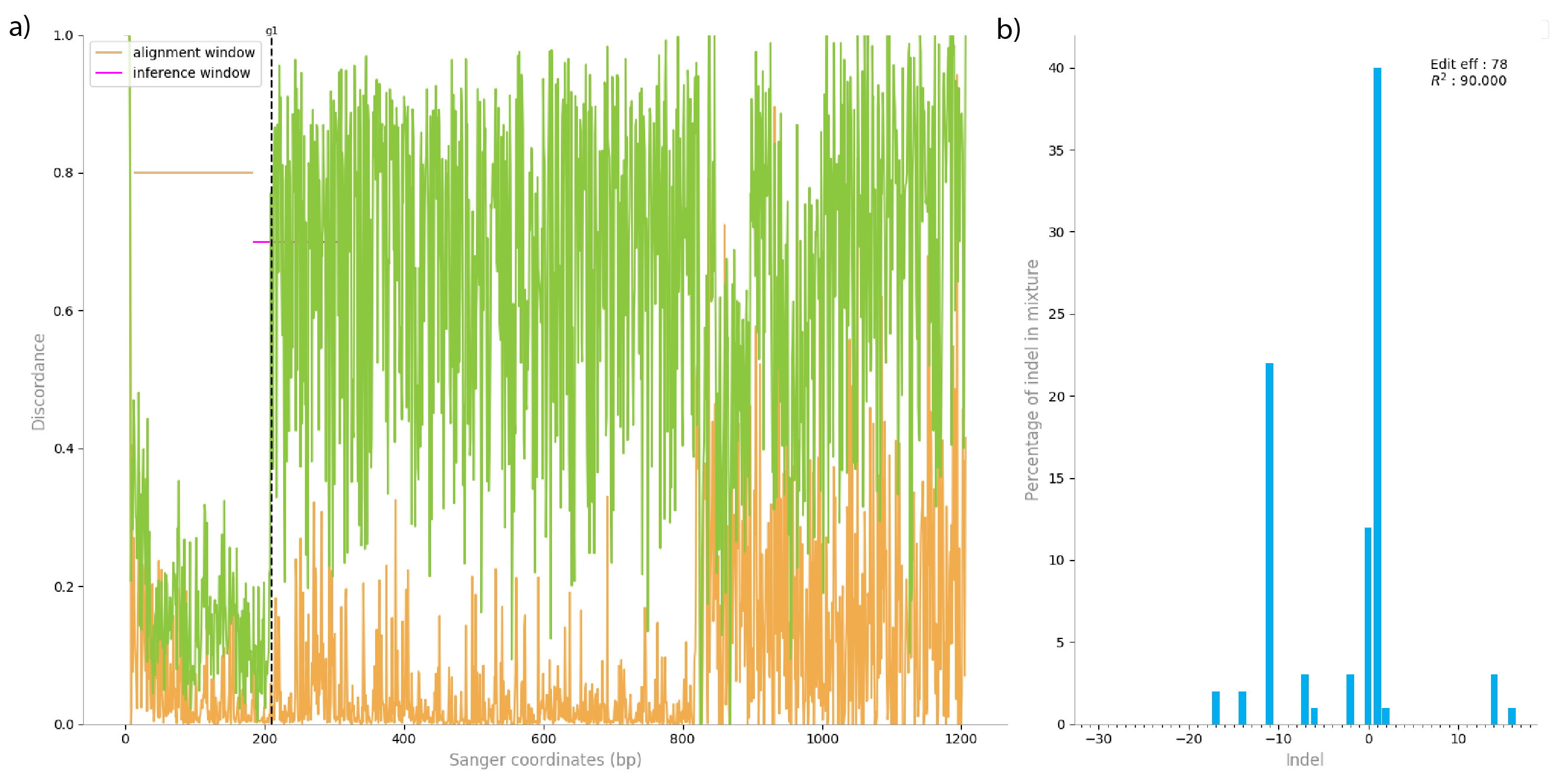


**Figure S6. INDELs in edited wild type CD34+ cells.** ICE analysis reporting a) the discordance between Sanger sequences of control and edited samples and b) the INDEL distribution pattern for the sgRNA-T89 targeting exon 1 of the NCF-2 locus around the cut site. The native ATG is at position -15 relative to the cut site and so the majority of INDELs are unlikely to directly impact the protein-coding sequence but may affect expression by disrupting regulatory regions.
