# Supplementary material for: Multidimensional Response Surface Methodology for the development of a gene editing protocol for p67phox-deficient Chronic Granulomatous Disease
Source: Hum Gene Ther. Author manuscript; Available in PMC 2024 Apr 16. (PMC7615834; doi:10.1089/hum.2023.114)
Supplement: Supplementary Methods [file EMS194183-supplement-Supplementary_Methods.docx]

**Supplementary Methods**

**AAV6 production**

30 x 150 mm cell culture dishes were seeded with 1.5 x 10^7^ HEK293-T cells each. After 18h, transfection was carried out with 25 kDa linear Polyethylenimine (PEI) (Polysciences, PA, USA). 2 mL of 1 mg/mL PEI was mixed with 26 mL DMEM and 0.22 µm-filtered. Separately, 225 µg of transfer plasmid and 660 µg of pDGM6 (Addgene 110660) were added to 33.65 mL of basal DMEM which was also filtered. The two solutions were mixed and incubated for 20 min at RT. This was then added to 555 mL fresh DMEM+FBS+P/S. The media for each dish was removed and replaced with 20.5 mL of the transfection media. After 24h, this was removed and replaced with 15 mL reduced-serum DMEM (1% FBS, 1x GlutaMax, 10 mM HEPES, 0.075% Sodium Bicarbonate, P/S) (Gibco). After a further 48 h, conditioned medium was collected and pooled from all plates, cleared by centrifugation at 1300 g, 10 min, 0.45 µm filtration, and then stored at 4 °C for up to 48 h. 10 mL PBS was added to each plate and cells were detached using scrapers. Cells were pooled, pelleted (1300 g, 10 min), and resuspended in 10 mL of PBS-MK (PBS + 1 mM MgCl_2_, 2.5 mM KCl) before freezing at -80 °C. The conditioned medium was pre-concentrated by adding 31.3 g% w/v Ammonium Sulfate, mixing thoroughly and leaving at RT for 10 min before incubating on ice for 30 min. Material was then pelleted (8300 g, 30 min, 4 °C) and resuspended in 10 mL PBS-MK. The cells were thawed and re-frozen three times before pelleting the cellular debris (3200 g, 15 min, 4 °C), and recovering the supernatant, which was then pooled with the pre-concentrated conditioned medium. Subsequently, 50 Units per mL Benzonase (NEB) and 0.5% w/v Deoxycholic acid were added and incubated for 60 min at 37 °C. The solution was again clarified by centrifugation (2400 g, 10 min, 4 °C) and passed through 0.22 µm syringe filters. 3.5 mL of AAV-containing medium was added to each of six ultracentrifuge tubes (Beckman 344059). Successive layers of iodixanol (optiprep) were underlaid using a 120 mm 12G needle and 3 mL syringe barrel as per Table S4.

Tubes were loaded onto a Sorval TH-641 swinging bucket rotor in Beckman Optima XE Ultracentrifuge and centrifuged at 273,799g (40,000 RPM) for 3 hours at 18C (Acc. Max, Dcn. 9). AAV were collected from the 40% fraction by piercing the side of the tube with a short 18G needle just below the 40/60% interface, angling the needle up into the 40% fraction and rotating the needle such that the aperture pointed upwards. Approximately 1 mL was withdrawn per tube, to avoid contamination from other fractions. AAV were washed and concentrated using 15 mL 100 kDa MWCO Amicon Ultra Centrifugal Filters (Merck, NJ, USA). A spin-filter was pre-wetted and washed by addition of 10 mL PBS to the top compartment and centrifuging at 1000 g, 3 min. AAV/Iodixanol was added to the top compartment and topped up to 12 mL with PBS. This was centrifuged at 2500-4000 g in increments of 3 min until volume reached 0.5 mL, before being topped up with PBS to 12 mL. This was repeated 3x. Finally, AAV were concentrated to the lowest volume achievable (~200 µL) before aliquoting and storage at -20 °C).

**Karyotyping**

Cells were treated with colcemid (100 ng/mL) for 30 minutes at 37 °C before pelleting at 150 x g for 8 minutes. Supernatant was aspirated and pellet dislodged by flicking before adding 4 mL pre-warmed 0.075 M KCl dropwise. Cells were incubated for 25 minutes at 37 °C. 4 mL of ice-cold 1:3 methanol:acetic acid fixative was added dropwise and inverted to mix, before centrifugation, removal of supernatant, and resuspension in 1.5 mL fixative. Karyotype of fixed cells was then analysed by Cell Guidance Systems Karyotype service.

**Western Blotting**

Frozen cell pellets were resuspended in RIPA buffer + complete mini protease inhibitor cocktail (Merck), incubated on ice for 15 min, then centrifuged at 16,000 g for 10 min before recovering supernatant, the concentration of which was quantified with a BCA protein assay kit (Pierce) according to manufacturer instructions. Proteins were denatured by addition of 4x LDS Sample Buffer and 10x Reducing Agent (NuPAGE, Thermo Fisher) and heating for 10 min at 75 °C. Proteins were separated on 4-12% NuPAGE gel (30 min at 40 V, 165 min at 100 V) before transfer to PVDF membranes using a mini trans-blot cell (BioRad). Membranes were blocked (1 h, RT) with 5% BSA in PBS-T (0.1% Tween), washed 3x 5 min with PBS-T, and incubated overnight with primary antibodies (Table S2) at 4 °C. Membranes were then washed again and incubated with secondary antibodies (1 h, RT) before washing and target detection with SuperSignal West Femto PLUS Chemiluminescent Substrate (Thermo Fisher) and imaged with the Syngene Genegnome XRQ.

**INDELs**

The indel frequency and size was measured by ICE Analysis (Synthego) of Sanger sequencing data of PCR amplicons of the cut site locus (see table S3) of unedited and sgRNA T89 RNP-edited wildtype CD34 cell genomic DNA.

**Supplementary Tables**

**Supplementary Table 1- RSO RNP calculations**

| **Electroporation** | **μg Cas9** | **μg gRNA:Cas9** | **Final Volume (μL)** | **μL Cas9** | **μL gRNA (5 μg/μL)** | **μL Cells** | **μL MaxCyte Buffer** |
| --- | --- | --- | --- | --- | --- | --- | --- |
| **A** | 10.00 | 0.30 | 25.00 | 1.00 | 0.60 | 15.00 | 8.40 |
| **B** | 6.59 | 0.60 | 25.00 | 0.66 | 0.79 | 15.00 | 8.55 |
| **C** | 10.00 | 0.90 | 25.00 | 1.00 | 1.80 | 15.00 | 7.20 |
| **D** | 15.00 | 0.10 | 25.00 | 1.50 | 0.29 | 15.00 | 8.21 |
| **E (1+2+3)** | 15.00 | 0.60 | 25.00 | 1.50 | 1.80 | 15.00 | 6.70 |
| **F** | 15.00 | 1.10 | 25.00 | 1.50 | 3.31 | 15.00 | 5.19 |
| **G** | 20.00 | 0.30 | 25.00 | 2.00 | 1.20 | 15.00 | 6.80 |
| **H** | 23.41 | 0.60 | 25.00 | 2.34 | 2.81 | 15.00 | 4.85 |
| **I** | 20.00 | 0.90 | 25.00 | 2.00 | 3.60 | 15.00 | 4.40 |
| **WT-E** | 0.00 | 0.00 | 25.00 | 0.00 | 0.00 | 15.00 | 10.00 |

**Supplementary Table 2- Antibodies**

| **Target** | **Species** | **Fluorophore** | **Dilution** | **Manufacturer** | **ID** |
| --- | --- | --- | --- | --- | --- |
| p67^phox^ | Rabbit | - | 1:50 (FACS), 1:1000 (WB) | Abcam | Ab109523 |
| GAPDH | Mouse | - | 1:1000 | Santa Crux Biotech | Sc-47724 |
| ECL anti-mouse | Sheep | - | 1:10000 | Cytiva | Nxa931v |
| ECL anti-rabbit | Donkey | - | 1:10000 | Cytiva | Na934v |
| Cy5 anti-rabbit | Goat | Cy5 | 1:500 | Invitrogen | A10523 |
| Cd11b | Rat | PE | 1:50 | BioLegend | 101208 |
| CD34 | Mouse | BV421 | 1:50 | BioLegend | 343610 |

**Supplementary Table 3-ddPCR primers and probes**

| Target | Forward Primer | Reverse Primer | Probe |
| --- | --- | --- | --- |
| coP67 Integration | CTGAGTAGGTGTCATTCTATTCTGGGG | AGGACAGCCTTCACAAAGGTGTTAA | FAM/ATGCCTGCT/ZEN/ATTCTCTTCCCAATCCTCCCCCTTGC/BHQ |
| coWAS Integration | TGGGAAGAGAATAGCAGGCATGC | CGTAAAGGCGGATGAAGTAGGACT | FAM/TGGCGCTGC/ZEN/CCCCTGGAGCT/BHQ |
| AAV ITR | GGAACCCCTAGTGATGGAGTT | CGGCCTCAGTGAGCGA | HEX/CACTCCCTC/ZEN/TCTGCGCGCTCG/BHQ |
| Albumin (0.5 kb) | TGGAGTGTTGCCCTTATTATGC | AGCTGCTGGTTCTCTTTCAC | HEX/CCTGTCATG/ZEN/CCCACACAAATCTCTCC/BHQ |
| Exon 3 Knockout locus | CAGGTAACTGATGACAATGCCTTGATG | GAGCCAGGAGTGAGCAGTATTCC | N/A |
| Exon 1 T89 Cut locus | CTACTCGCCCTCTCTCTCTCTG | AATCTCTTGAGCAGGCAAAGTC | N/A |

**Supplementary Table 4-ddPCR amplification conditions**

| Target | AAV2 ITR | coP67, coWAS, Albumin |
| --- | --- | --- |
| Pre-incubation | 10’, 95 °C | 10’, 95 °C |
| Cycle: Denaturation | 30 s, 95 °C (Ramp 2.5 °C/s) | 30 s, 95 °C (Ramp 1 °C/s) |
| Cycle: Annealing | 60 s, 60 °C (Ramp 2.5 °C/s) | 60 s, 60 °C (Ramp 1 °C/s) |
| Cycle: Extension | 15 s, 72 °C (Ramp 2.5 °C/s) | 2’, 72 °C (Ramp 1 °C/s) |
| Cycles | 40 | 49 |
| Inactivation | 10’, 95 °C | 10’, 95 °C |

**Supplementary Table 5-Iodixanol Gradient Formation**

| Iodixanol Concentration | Opti-prep (mL) | NaCl 5M (mL) | 5X PBS-MK buffer (mL) | Water (mL) | Phenol Red (µL) | Total Volume (mL) | Volume per tube (mL) | Colour |
| --- | --- | --- | --- | --- | --- | --- | --- | --- |
| 15% | 5 | 4 | 4 | 7 | 0 | 20 | 2.8 | Clear |
| 25% | 5.4 | 0 | 2.6 | 5 | 65 | 13.065 | 1.88 | Red |
| 40% | 8.16 | 0 | 2.4 | 1.44 | 0 | 12 | 1.55 | Clear |
| 60% | 10 | - | - | - | 50 | 10.05 | 1.55 | Yellow |
